# Salvage Radiotherapy after Radical Prostatectomy: Prediction of Biochemical Outcomes

**DOI:** 10.1371/journal.pone.0103574

**Published:** 2014-07-29

**Authors:** Ohseong Kwon, Ki Bom Kim, Young Ik Lee, Seok-Soo Byun, Jae-Sung Kim, Sang Eun Lee, Sung Kyu Hong

**Affiliations:** 1 Department of Urology, Seoul National University Bundang Hospital, Seongnam, Korea; 2 Department of Radiation Oncology, Seoul National University Bundang Hospital, Seongnam, Korea; University of Kentucky College of Medicine, United States of America

## Abstract

**Introduction:**

A significant proportion of patients undergoing salvage radiotherapy (RT) for biochemical recurrence (BCR) following radical prostatectomy (RP) may again experience BCR after salvage RT. Thus, we evaluated the clinical significances of different parameters on the biochemical outcome of RT in salvage setting.

**Methods:**

We reviewed the records of 212 patients who underwent salvage RT between November 2003 and December 2012 for BCR following primary RP. BCR-free survivals after salvage RT were estimated using the Kaplan–Meier method. Cox proportional hazard regression models were used to evaluate the impacts of clinicopathologic parameters on BCR following salvage RT.

**Results:**

The overall median follow-up duration was 63.5 months. The BCR-free survival rate after salvage RT was 58.2% at 5 years. Multivariate analysis showed that a pre-RT prostate-specific antigen (PSA) level of ≤0.5 ng/mL, a pre-RT PSA doubling time (PSADT) of >4.5 months, concomitant androgen deprivation therapy (ADT) with salvage RT, and a positive surgical margin were independent predictors of favorable biochemical outcomes after salvage RT (hazard ratios [HR] = 3.012, 1.132, 2.000, and 1.805, respectively, p = less than 0.001, 0.013, 0.005, and 0.036, respectively). In the early (pre-RT PSA ≤0.5 ng/mL) salvage RT setting, concomitant ADT administration was also shown to be significantly associated with higher risk of BCR-free survival following salvage RT (HR = 2.611, p = 0.038).

**Conclusion:**

Lower pre-RT PSA value, longer PSADT before salvage RT, concomitant ADT administration, and a positive surgical margin were significant predictors of favorable biochemical outcomes following salvage RT performed for BCR after primary RP.

## Introduction

Since Walsh's introduction of anatomical approach, radical prostatectomy (RP) has been widely performed as a definitive treatment for clinically localized prostate cancer (PCa). The technique of RP continues to be refined with emergence of robotic approach. However, published data have shown that up to 40% of patients undergoing RP may eventually experience biochemical failure with long-term follow-up [1], [2]. A considerable proportion of men undergoing RP would eventually need additional treatment. Certainly, postoperative biochemical recurrence (BCR) is associated with increased risk of distant metastasis and death from cancer. The median intervals from biochemical failure to distant metastasis and from metastasis to death have been reported to be about 8 years and 5 years, respectively [3].

For patients with suspected local failure following RP, external beam radiotherapy (RT) is a widely performed as salvage therapy. Although salvage RT for BCR following RP has been reported to decrease the risk of adverse outcome, a significant proportion of patients who underwent salvage RT demonstrate biochemical failure with 4- or 5-year biochemical disease-free survival rates after RT ranging from 46–60%. Accordingly, the identification of patients at higher risk of adverse outcome following salvage RT would be clinically important since such patients may benefit from adjuvant or more extensive forms of salvage therapy. Efforts to identify useful predictors, clinical or pathological, to select men at higher risk of failure after salvage RT would be critical for improving outcomes in salvage setting. Thus, we investigated the outcomes of patients who underwent salvage RT for biochemical failure following RP at our institution and sought to identify prognostic factors for such men.

## Materials and Methods

With the approval of the institutional review board at the Seoul National University Bundang Hospital (IRB No.: B-1404/248-108), we reviewed the records of 1893 PCa patients who underwent RP at our institution between November 2003 and December 2012. All data were analyzed anonymously. Of these patients, 363 (19.2%) experienced BCR. A total of 212 patients who underwent salvage RT were included in our study, and those who were administered neoadjuvant or adjuvant hormonal therapy were excluded from the study. None of the patients showed any clinical evidence of distant metastases before they underwent salvage RT. Of the 212 study participants, 124 (58.5%) received androgen deprivation therapy (ADT) concomitantly with the salvage RT. Prostate-specific antigen (PSA) levels were measured every 2–3 months during the first year, every 6 months in the second year, and then at least once per year following RP and salvage RT. Salvage RT was administered at a total dose of 60–70 Gy (median 66 Gy), delivered in daily fractional dose of 1.8–2.0 Gy. Target volume was defined adequately covering prostatic fossa and elective pelvic radiation of 45 Gy was used in 25% of all patients.

BCR after RP was defined as a PSA level of ≥0.2 ng/mL with 2 consecutive increases from a PSA value of <0.2 ng/mL, a PSA level that did not reduce to <0.2 ng/mL and increased twice, or the initiation of salvage therapy [4], [5]. The date on which BCR occurred was defined as the day on which the PSA level increased to ≥0.2 ng/mL or the day of the first follow-up visit if the PSA value did not decrease under 0.2 ng/mL [4]. BCR after salvage RT was defined as a PSA level that had increased to ≥0.2 ng/mL from the post-RT nadir confirmed by one more consecutive result. The time to BCR after salvage RT was defined as the period from salvage RT to the confirmatory PSA measurement [6]. The PSA doubling time (PSADT) was computed as previously reported [7], and was calculated using first order kinetics by dividing the natural log of 2 by the slope of the linear regression line of the natural log of PSA over time. Early salvage RT was defined as RT initiated when the PSA level was ≤0.5 ng/mL at the time of treatment [8].

We analyzed the following clinicopathologic factors: age, preoperative PSA levels, pathologic Gleason scores, pathologic stages, surgical margin statuses, postoperative PSA nadirs, pre-RT PSA levels, PSADTs before salvage RT, intervals from RP to salvage RT, concomitant ADT administration, and the BCR-free survival rates. All statistical analyses were performed using commercially available statistical software (IBM® SPSS® version 19.0, IBM, Armonk, New York, USA). Demographic and clinical parameters were compared using the chi-square test for categorical variables and Student's *t* test for continuous variables. The effects of different factors on the biochemical progression of the disease after salvage RT were analyzed using univariate and multivariate Cox proportional hazard regression models, and hazard ratios (HR) and 95% confidence intervals (CI) were calculated. The Kaplan–Meier method and the log-rank test were used to assess BCR-free survival rates. For all statistical analyses, a 2-tailed *P* value of <0.05 was considered statistically significant.

## Results

**Figure 1:**
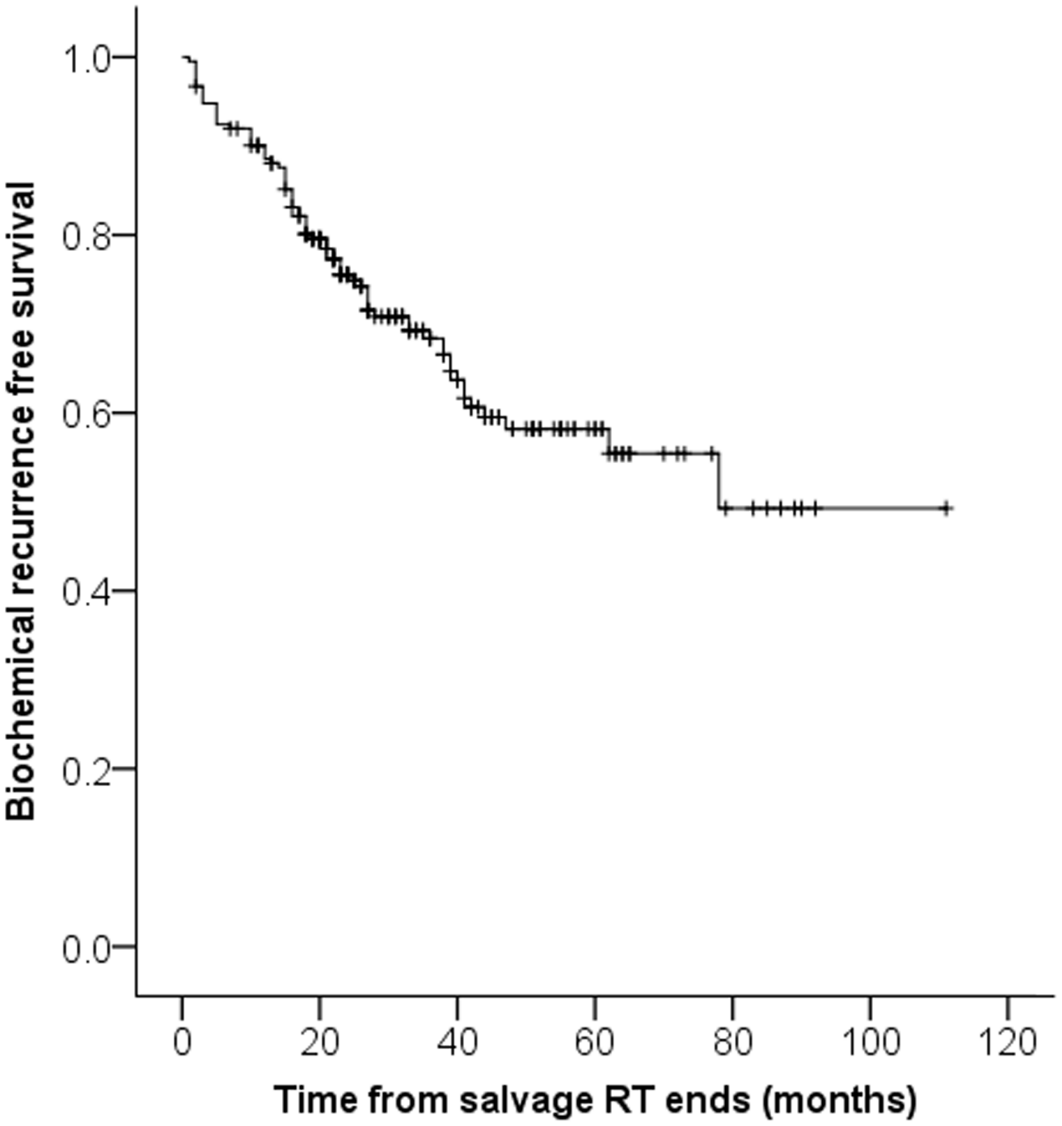
Kaplan-Meier estimates of overall biochemical recurrence free survival.

Of the 212 patients who underwent salvage RT, 71 (33.5%) experienced BCR after salvage RT. The patients' clinicopathologic parameters are shown in Table 1. The BCR-free survival rates at 1, 2, and 5 years for the 212 patients following salvage RT were 89.6%, 75.5%, and 58.2%, respectively (Figure 1). The median interval from salvage RT to BCR was 11.0 months, and the last BCR occurred 70 months after salvage RT. When the patients were stratified according to the occurrence of BCR following salvage RT, significant differences were found between the 2 groups in relation to the post-RP PSA nadirs (p = 0.035), pre-RT PSA levels (p = 0.004), pre-RT PSADTs (p = 0.008), RP to RT intervals (p = 0.007), rates of concomitant ADT administration (p = 0.038), and follow-up durations (p = 0.002).

**Table 1:** Characteristics of patients.

	Total (n = 212)	No BCR (n = 141)	BCR after salvage RT (n = 71)	P value
Median age, years (range)	66.0 (39–78)	66.0 (49–78)	66.0 (39–77)	0.817
Median preoperative PSA, ng/ml (range)	16.0 (1.8–86.6)	16.6 (1.8–86.6)	13.4 (2.3–53.7)	0.209
<10, n (%)	36 (17.0%)	21 (14.9%)	15 (21.1%)	0.254
≥10, n (%)	176 (83.0%)	120 (85.1%)	56 (78.9%)	
Gleason score				
≤7, n (%)	123 (58.0%)	87 (61.7%)	36 (50.7%)	0.126
≥8, n (%)	89 (42.0%)	54 (38.3%)	35 (49.3%)	
T Stage				
pT2a-c, n (%)	52 (24.5%)	40 (28.4%)	12 (16.9%)	0.155
pT3a, n (%)	70 (33.0%)	46 (32.6%)	24 (33.8%)	
pT3b-pT4, n (%)	90 (42.5%)	55 (39.0%)	35 (49.3%)	
Margin Positivity, n (%)	145 (68.4%)	98 (69.5%)	47 (66.2%)	0.625
Median pre-RT PSA, ng/mL (range)	0.46 (0.05–8.95)	0.36 (0.05–6.76)	0.84 (0.11–8.95)	0.004
≤0.5, n (%)	118 (55.7%)	95 (67.4%)	23 (32.4%)	<0.001
>0.5, n (%)	94 (44.3%)	46 (32.6%)	48 (67.6%)	
Median postoperative PSA nadir, ng/mL (range)	0.09 (0.001–7.47)	0.06 (0.001–7.47)	0.13 (0.001–6.05)	0.035
<0.1, n (%)	109 (51.4%)	80 (56.7%)	29 (40.8%)	0.029
≥0.1, n (%)	103 (48.6%)	61 (43.3%)	42 (59.2%)	
Median PSADT before salvage RT, months (range)	4.32 (0.45–208.64)	5.16 (0.67–208.64)	3.56 (0.45–17.33)	0.008
≤4.5, n (%)	113 (53.3%)	64 (45.4%)	49 (69.0%)	<0.001
>4.5, n (%)	99 (46.7%)	77 (54.6%)	22 (31.0%)	
ADT with RT, n (%)	124 (58.5%)	89 (63.1%)	35 (49.3%)	0.038
Median RP to RT interval, months (range)	11.0 (2–82)	13.0 (2–82)	6.0 (2–63)	0.007
Median radiation dose, Gy (range)	66.6 (60–75)	66.6 (60.0–75.0)	66.6 (60–70)	0.765
Median follow-up period, months (mean)	63.5 (63.2)	59.0 (59.3)	77.0 (70.1)	0.002

BCR, biochemical recurrence; PSA, prostate specific antigen; RT, radiotherapy; PSADT, PSA doubling time; ADT, androgen deprivation therapy; RP, radical prostatectomy.

Table 2 shows the Cox regression analyses of the different parameters thought to influence BCR-free survival. Univariate analysis showed that a higher pre-RT PSA level, a shorter pre-RT PSADT, and a shorter interval between RP to RT were significant predictive parameters for PSA progression. After dichotomizing the patients around the median values of these parameters, a pre-RT PSA level of ≤0.5 ng/mL and a pre-RT PSADT of >4.5 months were found to be significant predictive factors for longer BCR-free survival times after salvage RT. In addition, the concomitant use of ADT with RT was associated with longer BCR-free survival times. Multivariate analysis showed that a pre-RT PSA concentration of >0.5 ng/mL, a pre-RT PSADT of ≤4.5 months, salvage RT without ADT, and a negative surgical margin were independent predictors of PSA progression after salvage RT.

**Table 2:** Parameters influencing BCR free survival after salvage radiotherapy.

Parameter	Univariate analysis*P*-value (HR, 95% CI)	Multivariate analysis*P*-value (HR, 95% CI)
Pre-RT PSA ≤0.5 ng/mL	<0.001 (3.244, 1.966–5.353)	<0.001 (3.012, 1.724–5.262)
Pre-RT PSADT >4.5 months	0.003 (2.139, 1.292–3.542)	0.013 (1.132, 1.027–1.248)
Gleason score ≤7	0.057 (0.635, 0.398–1.014)	0.326 (1.142, 0.877–1.487)
Positive surgical margin	0.338 (1.273, 0.777–2.084)	0.036 (1.805, 1.041–3.125)
T stage (pT2a-c vs pT3a vs ≥ pT3b)		
pT2a-c	reference	reference
pT3a	0.224 (1.539, 0.768–3.084)	0.108 (0.693, 0.443–1.084)
≥ pT3b	0.048 (1.940, 1.004–3.748)	0.420 (1.161, 0.807–1.671)
Postoperative PSA nadir <0.1 ng/mL	0.098 (1.492, 0.928–2.399)	0.263 (1.417, 0.770–2.611.)
Pre-RP PSA ≥10 ng/mL	0.190 (1.465, 0.827–2.595)	—
Age ≤66 years	0.784 (1.067, 0.670–1.701)	—
Concomitant ADT	0.022 (1.725, 1.082–2.750)	0.005 (2.000, 1.238–3.226)
RP to RT interval >11.0 months	0.148 (1.419, 0.884–2.279)	—

BCR, biochemical recurrence; PSA, prostate specific antigen; RT, radiotherapy; PSADT, PSA doubling time; ADT, androgen deprivation therapy; RP, radical prostatectomy.

**Figure 2:**
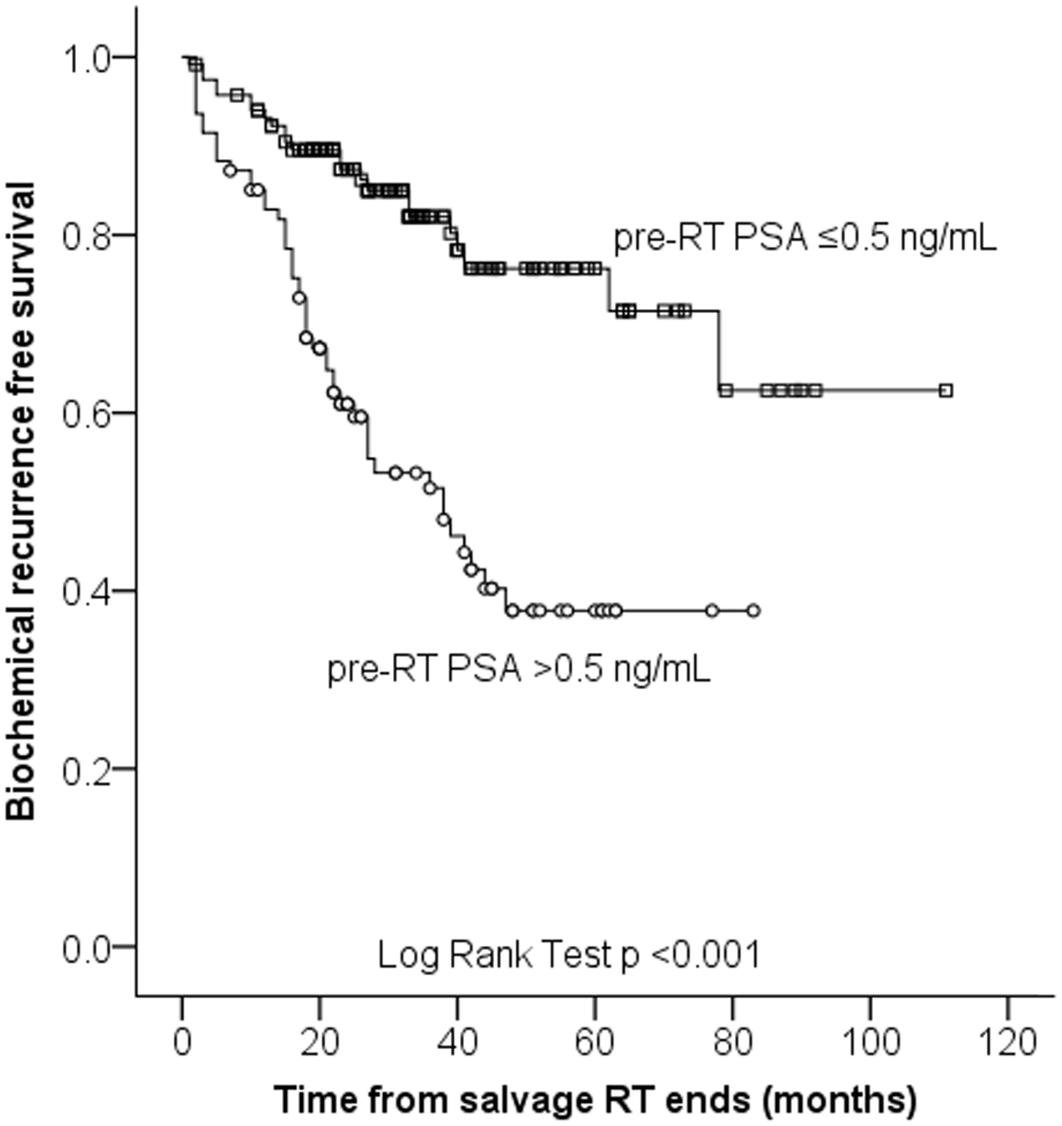
Kaplan-Meier estimates of biochemical recurrence free survival depending on pre-RT PSA.

Among the 212 patients, early salvage RT was administered to 118 patients (55.7%). In this subgroup, the BCR-free survival rates at 1, 2, and 5 years after early salvage RT were 92.8%, 87.6%, and 76.2%, respectively (Figure 2). The BCR free survival rates was significantly higher in early salvage RT group than those of overall patients including early salvage RT group (p = 0.007). Multivariate analysis showed that the administration of concomitant ADT was a significant predictor of PSA progression within the early salvage RT group (HR 2.611, 95% CI 1.055–6.452, p = 0.038) in Table 3.

**Table 3:** Parameters influencing BCR free survival after early salvage radiotherapy.

Parameter	Univariate analysis*P*-value (HR, 95% CI)	Multivariate analysis*P*-value (HR, 95% CI)
Pre-RT PSA (continuous)	0.416 (1.044, 0.968–1.126)	0.400 (4.711, 0.128–5.836)
Pre-RT PSADT >4.5 months	0.711 (1.168, 0.514–2.653)	0.862 (1.086, 0.428–2.755)
Gleason score ≤7	0.699 (1.186, 0.500–2.813)	0.950 (0.969, 0.358–2.623)
Positive surgical margin	0.734 (1.176, 0.460–3.012)	0.356 (1.597, 0.592–4.310)
T stage (pT2a-c vs pT3a vs ≥ pT3b)		
pT2a-c	Reference	Reference
pT3a	0.717 (0.824, 0.290–2.342)	0.883 (1.088, 0.351–3.375)
≥ pT3b	0.550 (0.748, 0.288–1.941)	0.554 (1.429, 0.439–4.654)
Postoperative PSA nadir <0.1 ng/mL	0.605 (1.247, 0.540–2.881)	0.540 (1.192, 0.679–2.094)
Pre-RP PSA ≥10 ng/mL	0.092 (1.036, 0.994–1.080)	—
Age ≤66 years	0.392 (1.028, 0.965–1.096)	—
Concomitant ADT	0.055 (2.280, 0.981–5.291)	0.038 (2.611, 1.055–6.452)
RP to RT interval >11.0 months	0.255 (1.019, 0.986–1.055)	—

## Discussion

In this study, the 5-year BCR-free survival rate was 58.2%, a rate that was comparable with other studies [9]. Our data show that predictive factors for BCR after salvage RT include a pre-RT PSA level of >0.5 ng/mL, a pre-RT PSADT of ≤4.5 months, salvage RT without concomitant ADT, and a negative surgical margin. The 5-year BCR-free survival rate for the early salvage RT group was 76.2%, which was significantly higher than that of the patients outside this subgroup. Analysis of the early salvage RT patient subgroup showed that concomitant ADT was the only factor that reduced BCR after RT. Overall our findings suggest that salvage RT performed early for presumed-local recurrence with concomitant ADT would be most effective.

A large systemic review of 41 retrospective studies on salvage RT following RP demonstrated that PSA level before salvage RT was significantly associated with BCR -free survival [9]. Overall there was an average of 2.6% loss of BCR- free survival for each incremental 0.1 ng/ml of PSA at the time of salvage RT. BCR- free survival following salvage RT approached 64% when PSA was <0.2 ng/ml before salvage RT. Looking at the literature, clinical significance of pre-RT PSA level had also been demonstrated by the findings from matched-control analyses of adjuvant versus salvage RT. In such studies, adjuvant RT group who had PSA <0.2 ng/ml was observed to have about 20% higher relapse-free survival compared with salvage RT group whose PSA was greater amongst the patients with uniformly matched high risk pathologic features [10]. In our study, we also observed that patients with lower PSA level before salvage RT had better outcome. Several studies have shown better outcome with PSA level before RT below a certain threshold, typically at 0.5, 1.0, or 2.0 ng/mL [11], [12]. Meanwhile, controversy still continues on how high that threshold could be. According to an exponential biologic model from the aforementioned systemic review, it was suggested that a continuously progressive loss of tumor control occurred with PSA elevation before salvage RT. This hypothesis would be consistent with superior effectiveness of RT for lesser microscopic tumor burden. The effectiveness of salvage RT relies heavily on the PSA level during treatment [13], [14]. Consequently, salvage RT should be initiated as soon as possible after PSA recurrence. Karlin et al. suggested that salvage RT should be initiated as soon as PSA recurrence becomes evident, even at PSA levels of ≤0.33 ng/mL in patients with a Gleason score of ≥8 [15]. A current guideline proposes a PSA level of ≤0.5 ng/mL as the cutoff value for salvage RT.^7^ Furthermore, this study showed the favorable effect of early salvage RT on BCR-free survival. Briganti et al. reported an overall 5-year BCR-free survival rate of 73.4%, which is similar to the 5-year BCR-free survival rate of 76.2% determined in the current study [16].

The administration of concomitant ADT may be a treatment option for patients with a higher probability of relapse after RT. However, the role played by concomitant ADT in slowing cancer progression after salvage RT remains a topic of debate. Furthermore, ADT may cause cardiovascular comorbidities and negatively impact a patient's quality of life [17], [18]. ADT may also exacerbate the local adverse effects that are associated with RT. Trock et al. reported that adding ADT to salvage RT did not increase PCa-specific survival [11]. Salvage RT alone or salvage RT plus ADT did not show a significant difference between each other (p = 0.98), though both group showed significant increase in PCa-specific survival compared with no salvage therapy (p<0.001) [11]. On the contrary, Soto et al. suggested that ADT administered concurrently with salvage RT was beneficial in reducing BCR after RT in high-risk patients (HR 0.65, p = 0.046) [19]. The overall patient analysis in the current study showed that ADT administered concurrently with salvage RT was a predictive parameter for favorable patient outcomes. In the analysis of the early salvage RT patient subgroup, ADT administered concurrently was the only predictive parameter for positive patient outcomes. Meanwhile, although concomitant ADT was observed to be beneficial in the salvage RT setting, our findings should be interpreted with caution. First, the duration or regimen for ADT during salvage RT varied among our patient cohort. The median duration of ADT administration was 15 months, but the most appropriate duration of ADT usage has yet to be determined through further studies. The timing of ADT in patients with BCR and the concomitant use of ADT with salvage RT remain still controversial [20]. In a study by Umezawa et al., ADT was administrated before and/or during salvage RT for a median length of 6 months (range, 1–18 months) in 29 patients and 11 of 29 received ADT for a median length of 12 months [21]. Moreover, ADT tends to be used for approximately 6 months as an adjuvant therapy, and for up to 3 years in patients with high-risk PCa [19]. Further studies are needed to estimate the optimal duration of ADT administration. A prospective study would be extremely valuable to determine the efficacy of concomitant ADT on BCR after salvage RT to establish the risk of comorbidities and determine any impact on the quality of life.

Positive surgical margins indicate the presence of remnant tumor in the surgical beds and independently predict BCR after RP [22]. However, their impact on systemic cancer progression remains a topic of debate [23]. A positive surgical margin that may result from limited experience of a surgical technique might represent local disease. Stephenson et al reported that patients with positive surgical margins are more likely to have better prognoses after salvage RT than those with negative margins (HR 1.7, 95% CI 1.4–2.5, p<0.001) [24]. Also Kinoshita et al. reported that a negative surgical margin was an independent predictor of BCR-free survival after salvage RT (HR 0.28, 95% CI 0.12–0.70, p = 0.006) [25]. The overall analysis of the patients in the present study suggested that a positive surgical margin was a statistically significant predictor of longer BCR-free survival after salvage RT. Indeed, the response to salvage RT has generally been reported to be better in patients with positive surgical margins [26]. However, some have reported to the contrary. Briganti et al. suggested that a positive surgical margin may be a predictor of increased risk of BCR after early salvage RT (HR 1.62, 95% CI 1.10–2.39, p = 0.01) [16]. Meanwhile, a study by Buskirk et al. suggested that the status of the surgical margin did not have significant impacts on the BCR-free survival rate or on the overall survival rate after RT (p = 0.89) [27]. Overall more studies are needed to confirm the impact of surgical margin status on disease progression following salvage RT.

The PSADT is another commonly used parameter in clinical practice, as it indicates the dynamics of the PSA levels. The PSADT is valuable in identifying appropriate patients for salvage treatments after BCR [28]. D'Amico et al. proposed that a PSADT of <3 months was statistically related to the risk of death from PCa [29], and the authors recommended a PSADT of <3 months as an indicator for the initiation of systemic treatment. Zhou et al. emphasized the use of the PSADT as a suggestive prognostic parameter after RP and radical RT [30]. A post-RP PSADT and a post-RT PSADT of <3 months were significantly associated with PCa-specific mortality. In addition, from their study Trock et al. suggested that the survival benefit after salvage RT is most beneficial in men with a PSADT of <6 months who are administered treatment within 2 years of the PSA levels increasing [11]. No definitive cutoff value exists for PSADT that enables the prediction of outcomes after salvage RT. Although a short PSADT is considered a relative risk factor for disease progression, Lohm et al. explained that in their study, patients with short PSADTs had shown low cancer-specific mortalities probably because of the higher sensitivity of the tumor tissue to salvage RT [31]. In this study, patients with short PSADTs showed higher BCR rates, which concurs with previous studies. In our study, when patients were dichotomized based on a PSADT of 4.5 months, which was an approximation of the median value, a PSADT of <4.5 months was a predictor of BCR after salvage RT.

Our study was limited by its retrospective nature and a relatively small number of subjects. The differences in the follow-up periods between patients without BCR and those with BCR after salvage RT (median follow-up period, 59.0 months and 77.0 months, respectively, p = 0.002) represented another weakness in this study. However, BCR occurred at a median follow-up time point of 17.0 months, and no events occurred after 62 months. Furthermore, since there were only 2 recurrences after the 59-month follow-up time point, the difference between the groups in relation to the follow-up period is not considered to have caused an appreciable comparison bias. Our data support early initiation of salvage RT and concomitant use of ADT, but further study is needed to define appropriate duration of ADT and to select favorable candidates for concomitant ADT.

## Conclusions

In this study, we observed that the pre-RT PSA level, the PSADT before salvage RT, concomitant ADT administration, and surgical margin status were significant predictors of favorable outcomes among patients who receive salvage RT for BCR after primary RP. Furthermore, early salvage RT initiated before the PSA level increased to 0.5 ng/mL substantially reduced BCR in patients. Overall analysis of the patients in the current study showed that concurrent administration of ADT with RT was a predictive parameter for favorable patient outcomes. We believe that our findings would contribute to the selection of appropriate candidates and optimize the outcome of salvage RT.
